# Exchange transfusion can be life-saving in severe propanil poisoning: a case report

**DOI:** 10.1186/1756-0500-7-700

**Published:** 2014-10-08

**Authors:** Priyanga Ranasinghe, Shani Apsara Dilrukshi, Inoshi Atukorala, Prasad Katulanda, Ariaranee Gnanathasan

**Affiliations:** Department of Pharmacology, Faculty of Medicine, University of Colombo, Colombo, Sri Lanka; University Medical Unit, National Hospital of Sri Lanka, Colombo, Sri Lanka; Department of Clinical Medicine, Faculty of Medicine, University of Colombo, Colombo, Sri Lanka

**Keywords:** Propanil, Poisoning, Exchange transfusion, Sri Lanka

## Abstract

**Background:**

Propanil is an important cause of herbicide poisoning in Sri Lanka, accounting for about 2% of all cases of self-poisoning. The outcome is extremely poor when the poisoning is severe and current medical care is of limited efficacy. Death usually occurs due to the severe and prolonged methaemoglobinaemia. We describe a case of severe Propanil poisoning, successfully treated by exchange transfusion at a tertiary care hospital in Sri Lanka.

**Case presentation:**

A 17-year old Sri Lankan male (body weight – 42 kg), presented to a local hospital 1 hour after self-ingestion of nearly 500 ml (4.3 g/kg) of liquid Propanil (concentration – 360 g/l). On admission he had dizziness and peripheral cyanosis. He was given intravenous methylene blue (1 mg/kg) within one hour of admission, which was repeated subsequently due to minimal response. The next day morning, (18 hours after poisoning) the patient was transferred to the National Hospital of Sri Lanka (NHSL) for further management. On admission to NHSL, he was drowsy and confused, had a shallow respiratory effort and marked central and peripheral cyanosis. Respiratory rate was 20/min, with a pulse-oximetry of 77% on room air. The arterial blood gas analysis was as follows; pH–7.24, HCO3^−^–12 mmol/l, pCO2–28 mmHg, pO2–239 mmHg and O2 saturation–100%. Exchange transfusion was commenced within two hours of admission to NHSL. A dramatic improvement in oxygen saturation was observed immediately afterwards, with the saturation in pulse-oximetry rising to >95%. The level of consciousness and respiratory effort also improved. He was discharged subsequently 8 days after the initial poisoning.

**Conclusion:**

Propanil has potential to produce severe life threatening clinical manifestations, despite categorization as a herbicide with low toxicity. In cases of severe poisoning, exchange transfusion may be life saving. Since methylene blue, intensive care and exchange transfusion facilities are also not readily available in local hospitals, which frequently encounter cases of severe Propanil poisoning, early transfer of patients to tertiary care hospitals should be considered. Exchange transfusion may be helpful even in late stages in patients with severe poisoning.

## Background

Propanil (3,4-dichloropropionanilide) is an important cause of herbicide poisoning. It is a widely used selective acetanilide [1]. It is possibly the most extensively used herbicide in rice cultivation worldwide [2]. However, available literature suggests that Propanil is a rare cause of poisoning. Ingestion leading to severe morbidity and death has been reported with Propanil, particularly in South Asian countries such as Sri Lanka [1, 3]. It is estimated that Propanil accounts for about 2% of all cases of self-poisoning admitted for hospital care in Sri Lanka, where it is the second most lethal herbicide after paraquat [1, 4]. Although rare, the outcome is extremely poor when the poisoning is severe and current medical care in severe poisoning is of limited efficacy [1].

Propanil and its major metabolite, 3, 4-dichloroanilide induces the conversion of Fe^2+^ in haemoglobin to Fe^3+^, forming methaemoglobin [1]. This results in the lowering of the oxygen carrying capacity of blood and tissue hypoxia. Clinical features include; gastrointestinal irritation causing abdominal pain, nausea and vomiting, central and peripheral cyanosis due to methaemoglobinaemia, headache, dizziness, stupor, convulsions, bradycardia, hypotension, respiratory depression, acidosis and haemolytic anaemia [5]. Death usually occurs due to the severe and prolonged methaemoglobinaemia [1]. We describe a case of severe Propanil poisoning, successfully treated by exchange transfusion at a tertiary care hospital in Sri Lanka.

## Case presentation

A 17**-**year**-**old Sri Lankan male, presented to a local hospital after self-ingestion of nearly 500 ml of liquid Propanil (360 g/l), in an attempt at deliberate self harm after a domestic dispute. The family had unsuccessfully attempted induced emesis at home with soap water. He was brought to the nearby local hospital one hour after ingestion. On admission the patient had dizziness, peripheral cyanosis and burning abdominal pain. The local hospital induced emesis using NaHCO_3._ However gastric lavage was not done and activated charcoal was not given. Subsequently, the patient developed marked respiratory difficulty, central and peripheral cyanosis and drowsiness. He was given intravenous methylene blue (1 mg/kg) within one hour of admission. Due to minimal improvement in respiratory difficulty, central and peripheral cyanosis and drowsiness methylene blue infusion was repeated one hour after the initial dose. In addition oral ascorbic acid (2 g three times per day) was given together with IV fluids and supplementary O_2_ via face mask, while vital parameters were also monitored. The next morning, nearly 18 hours after ingestion, the patient was transferred to the National Hospital of Sri Lanka, the premier tertiary care hospital in the country. The reasons for transfer were further deterioration, non-availability of further IV methylene blue preparations and the lack of intensive care and exchange transfusion facilities at the local hospital.

The patient arrived at the National Hospital of Sri Lanka 22 hours after ingestion of the poison, including 4 hours for transport from the local hospital. On admission to the National Hospital of Sri Lanka, he was drowsy and confused, but arousable. He had a shallow respiratory effort and marked central and peripheral cyanosis. On admission the heart rate was 120 beats/min with regular rhythm, blood pressure 100/60 mmHg. The respiratory rate was 20 breaths/min, with a pulse oximetry of 77% on room air and clear lungs on auscultation. The abdomen was soft, with epigastric tenderness. Neurological examination was unremarkable. The arterial blood gas analysis was as follows; pH – 7.24, HCO^3−^ – 12 mmol/l, pCO_2_ – 28 mmHg, pO_2_: 239 mmHg and O_2_ saturation – 100%. Intensified monitoring was commenced and supportive care was provided with IV fluids and supplementary O_2_ (6 l/min) via face mask. Although the patient showed mild improvement in his respiratory status, there was no change in his cyanosis yet and pulse oximetry demonstrated an oxygen saturation fluctuating between 75%-85%. Serum methaemoglobin levels were not measured due to the non-availability of facilities. The patient’s clinical condition continued to deteriorate with worsening drowsiness, increased respiratory rate (30 breaths/min) and tachycardia (130 beats/min), so it was decided it to commence on exchange transfusion without delay.

Exchange transfusion was performed within two hours of admission to the tertiary care hospital, via a femoral catheter. The total blood volume removed was 1875 ml, which was replaced with 1780 ml of packed red cells and 800 ml of normal saline (body weight – 42 kg, estimated blood volume – 3150 ml). A dramatic improvement in oxygen saturation was observed immediately after the procedure with the saturation rising to more than 95%, which remained stable until discharge. The second day after the exchange transfusion he exhibited marked improvement in the peripheral and central cyanosis. The level of consciousness, respiratory effort (18 breaths/min) and heart rate (80 beats/min) also improved. The patient developed a mild haemolysis on the second day after exchange transfusion, with indirect hyper-bilirubinaemia (total bilirubin – 3.9 mg/dl, indirect – 2.4 mg/dl and direct – 1.5 mg/dl), increased LDH level (900 iu/l) a blood picture demonstrating polychromatic red cells, and urinalysis showing increased urobilinogen levels. It improved gradually and the results of the serial full blood count reports are summarized in Table 1. His electrocardiogram, blood coagulation profile, renal and liver function tests remained normal throughout the hospital stay. He was discharged subsequently 8 days after the initial poisoning.

**Table 1:** **A summary of the patients full blood count from admission to discharge**

	1 ^st^Day (pre-ET)	1 ^st^Day (post-ET)	2 ^nd^Day	3 ^rd^Day	4 ^th^Day	5 ^th^Day	6 ^th^Day	8 ^th^Day
White cell count (μl)	10,380	10,790	9,900	8,900	7,600	6,840	6,400	6,500
Neutrophils (%)	74.7	68.7	63.0	64.9	66.6	64.2	64.0	64.2
Lymphocytes (%)	14.9	21.1	24.5	20.2	17.8	18.9	17.9	18.0
Monocytes (%)	6.6	6.4	8.4	7.5	8.3	7.6	11.5	11.0
Eosinophils (%)	3.6	4.0	1.3	3.7	5.3	5.2	5.2	5.1
Basophils (%)	0.2	0.2	2.8	3.7	2.0	4.1	1.4	1.9
Haemoglobin (g/dl)	14.7	13.0	14.6	14.6	13.6	14.0	12.8	13.6
Platelets (μl)	135,000	180,000	107,000	96,000	95,000	92,000	101,000	151,000

ET – Exchange Transfusion.

## Discussion

Propanil is a highly effective herbicide from acetanilide group, it is sold in at least 20 different brand names, mostly as 36% solutions [4]. It is often described in the toxicology literature as being a mild form of poison, with limited toxicity [6]. The current case demonstrates that it can be a severe form of self poisoning, especially after heavy ingestion. The lethal dose in man is probably as little as 10 ml of undiluted compound and ingestion of more than 200 ml of diluted Propanil is usually considered as a severe poisoning [5]. The onset of the development of methaemoglobinaemia is said to be proportional to the level of toxicity [4]. Clinical toxicity is characterised by cyanosis, acidosis and progressive end-organ dysfunction which are consistent with severe and prolonged methaemoglobinaemia [1]. Methaemoglobinaemia occurs due to bioconversion of propanil to 3,4-dichlorophenylhydroxylamine, which is co-oxidised with oxyhaemoglobin (Fe2+) in erythrocytes to the ferric state (Fe3+) [7]. This leads to end-organ dysfunction, manifesting particularly as central nervous system depression, hypotension and acidosis [8]. However, toxic mechanisms other than methaemoglobinaemia may contribute to clinical outcomes via lipoperoxidation, myelotoxicity, and immune dysfunction [1]. The elimination half-life of Propanil in humans is about 3.2 hours, however, the concentration of its major metabolite 3,4-dichloroaniline (DCA) is generally higher, more persistent and more variable than Propanil [1]. This is due to DCA having a longer half-life and being slightly less lipophilic than propanil and therefore likely to have a smaller volume of distribution [1]. Given that poisoning manifests early post-ingestion and yet the time to death is usually greater than 24 hours there are ample opportunities for interventions that prevent death [1]. Our patient presented with a rapid onset of methaemoglobinaemia, central nervous system depression, respiratory distress and haemolysis, which probably occurred rapidly due to the accelerated formation of methaemoglobin following ingestion of large quantities of Propanil. Haemolysis occurs in nearly 1/3rd of the patients with Propanil poisoning, possibly due to direct oxidant damage to red cells from the poison [9].

Methylene blue is considered as the drug of choice in methaemoglobinaemia, it is given in doses of 1-2 mg/kg IV [10]. It increases the rate of conversion to of methaemoglobin to haemoglobin. However, the condition of our patient continuously deteriorated, despite repeated doses of methylene blue soon after admission. However, the dose used in the present patient could have been inadequate, and IV methylene blue was not readily available in the hospital to administer repeated doses. Hence he was transferred to a tertiary care hospital for the commencement of exchange transfusion. Exchange transfusion replaces methaemoglobin and may also remove the remaining poison [4]. However, the efficiency of this varies inversely with the proportion of poison located outside the vascular compartment, which depends on the volume of distribution. The volume of distribution is high since both propanil and its major metabolite 3,4-dichloroaniline (DCA) are lipophilic [1]. Hence, from this it may be questioned whether the poison load will be markedly lowered by exchange transfusions [1]. Exchange transfusion can improve oxygen delivery by donation of erythrocytes, although their function is temporarily impaired post-transfusion due to depletion of 2,3-diphosphoglycerate (2,3-DPG) during storage [11]. Exchange transfusions are usually performed when patients fail to improve with the initial administration of methylene blue and in severe poisoning it may be a life saving measure [5]. Complications of exchange transfusion are mostly those related to any blood transfusion, such as transmission of blood-borne infections, electrolyte imbalance and transfusion reactions, in addition venous catheter site infections can also occur [12].

Our patient’s condition rapidly improved following exchange transfusion. Considering the rapid improvement, we suggest that exchange transfusions may be life saving, in cases of severe Propanil poisoning and recommend early commencement. In addition studies from Sri Lanka have shown that management difficulties in Propanil poisoning arise due to the lack of IV methylene blue, inability to measure methaemoglobin levels and paucity of intensive care unit beds, especially in the setting of smaller regional hospitals [3]. Furthermore, facilities for exchange transfusion are also not readily available in these hospitals. Hence in cases of severe poisoning we recommend early transfer of patients to tertiary care hospitals with facilities for exchange transfusion, which can be life saving as in the case of our patient. Exchange transfusions were commenced in this patient nearly 28 hours after the ingestion of the poison, demonstrating that it can be helpful even in late stages in patients with severe Propanil poisoning. However further studies are required to determine the best timing for the exchange transfusion. Furthermore, since it is a relatively common and a readily treatable cause of poisoning, there is also an urgent need to formulate evidence based management guidelines. In addition since Propanil poisoning is relatively common in Sri Lanka we also recommend that biomarkers to determine severity and response to treatment should be measured, including changes in methaemoglobinaemia should be measured and facilities should be made available at least in tertiary care hospitals.

## Conclusion

Propanil despite categorization as a herbicide with low toxicity, has potential to produce severe life threatening clinical manifestations. In cases of severe Propanil poisoning, exchange transfusions may be life saving, and probably requires early commencement. Since facilities for exchange transfusion are also not readily available in local hospitals, which frequently encounter cases of severe Propanil poisoning, early transfer of patients to tertiary care hospitals should be considered.

## Consent

Written informed consent was obtained from the patient’s legal guardian for publication of this case report and any accompanying images. A copy of the written consent is available for review by the Editor-in-Chief of this journal.

## Abbreviations

IV: Intravenous.
